# New Measurable Indicator for Tuberculosis Case Detection

**DOI:** 10.3201/eid1009.040349

**Published:** 2004-09

**Authors:** Martien W. Borgdorff

**Affiliations:** * KNCV Tuberculosis Foundation, The Hague, the Netherlands;; † University of Amsterdam, Amsterdam, the Netherlands

**Keywords:** tuberculosis, control, impact, case detection, perspective

## Abstract

The World Health Organization's goal for tuberculosis (TB) control is to detect 70% of new, smear-positive TB cases and cure 85% of these cases. The case detection rate is the number of reported cases per 100,000 persons per year divided by the estimated incidence rate per 100,000 per year. TB incidence is uncertain and not measured but estimated; therefore, the case detection rate is uncertain. This article proposes a new indicator to assess case detection: the patient diagnostic rate. The patient diagnostic rate is the rate at which prevalent cases are detected by control programs and can be measured as the number of reported cases per 100,000 persons per year divided by the prevalence per 100,000. Prevalence can be measured directly through national prevalence surveys. Conducting prevalence surveys at 5- to 10-year intervals would allow countries with high rates of disease to determine their case detection performance by using the patient diagnostic rate and determine the effect of control measures.

## Abstract 2

Conducting prevalence surveys at 5- to 10-year intervals would allow countries to determine their case detection performance.

Reversing global tuberculosis (TB) incidence by 2015 is included in the Millennium Development Goals (1). Prevalence and death rates (indicator 23) and the proportion of cases detected and cured under a directly observed treatment strategy (DOTS) (indicator 24) are used to measure progress towards this goal. For indicator 24, the World Health Organization (WHO) has formulated the following goals: a case detection rate of 70% and a cure rate of 85% (2,3). If both targets are achieved, the effect on TB transmission will be considerable (3,4).

WHO defines the cure rate as the proportion of new cases of smear-positive TB that were cured through treatment; this rate is routinely measured by treatment registers. The case detection rate is the proportion of incident smear-positive TB cases detected through a TB program. The case detection rate is measured as the notification rate of new cases of smear-positive TB divided by the estimated incidence rate.

Incidence is estimated by using various sources of information (5,6). An important element in these estimates is the proposed relationship between the incidence of TB and the annual risk for TB infection. Styblo estimated that, in the absence of control, a 1% (i.e., 1,000/100,000) annual risk for infection would correspond with an incidence of new cases of smear-positive TB of approximately 50 per 100,000 (7,8). In other words, in the absence of control measures, 50 cases would generate 1,000 infections; i.e., the average patient with a new case of smear-positive TB would generate approximately 20 infections over time. The annual risk for infection is measured imprecisely through tuberculin surveys; problems include cross-reactions caused by *Mycobacterium bovis* bacillus Calmette-Guérin vaccination and environmental mycobacteria. The relationship between risk for infection and incidence varies, depending on the quality of the control measures and the role of HIV infection (9). Deriving incidence from prevalence and the average duration of disease (6) also gives uncertain results, in particular because the duration of disease cannot be measured with precision. Deriving incidence from the number of TB deaths and estimated TB case death rates (6) also gives uncertain results because ascertaining cause of death is incomplete in most countries with a high rate of TB, and TB case death rates vary, since they depend on the quality of treatment and are strongly influenced by HIV co-infection (6). Therefore, incidence estimates are particularly uncertain in sub-Saharan Africa, which has the highest per capita TB incidence and prevalence of HIV infection in the world (5,6).

To measure the incidence of new cases of smear-positive TB directly, one would require at least two prevalence surveys, e.g., 1 year apart, as well as a surveillance mechanism to detect incident cases in patients dying or emigrating out between the first and second survey. Moreover, correct identification of persons with TB is needed to link results of the second survey to the first. If the time between surveys is reduced, this reduces the bias of patients dying or moving out, but the number of incident cases will be smaller, reducing precision. Direct measurement is thus costly and complicated, and no country is currently applying this method. As a result, the incidence of new cases of smear-positive TB is uncertain, and TB programs do not know whether they are reaching the case detection rate goal. This problem affects low-income countries with high rates of TB in particular, since these countries tend to have inadequate case detection and reporting systems.

These measurement problems are important because the effect of TB programs depends on their success in detecting cases. This article proposes an alternative indicator to measure TB case detection. This indicator does not directly measure the proportion of cases detected but the speed at which they are detected.

## New Indicator: Patient Diagnostic Rate

Since the case detection rate is estimated indirectly and is uncertain, another indicator that can be measured more directly would be desirable. This indicator is the rate at which prevalent case-patients are recruited by TB programs, referred to here as the patient diagnostic rate. In practice, this indicator can be measured as follows: the number of newly reported cases (i.e., never treated) of smear-positive TB per 100,000 population per year (notification rate) divided by the prevalence of new cases of smear-positive TB per 100,000 population. The numerator is obtained from surveillance data and the denominator from a prevalence survey. The denominator represents the population at risk for case detection, the numerator those actually detected. At present, the proposal is to restrict patient diagnostic rate to smear-positive cases because smear microscopy is currently the most widely applied tool to confirm TB in countries with high rates of disease. The proposal is restricted to new cases, since this best captures the effects of case detection. The prevalence of previously treated TB depends strongly on the cure rate. Patient diagnostic rates in countries conducting and reporting a prevalence survey during the past decade are presented in the Table.

A more refined estimate of patient diagnostic rate may be obtained by stratification for important variables that are recorded routinely, such as age, sex, urban versus rural areas, and DOTS versus non-DOTS areas. DOTS areas are defined as those that have adopted the WHO TB control strategy. Such stratification may help identify TB priorities for strengthening case finding and assess the effect of DOTS. In countries with a high prevalence of HIV infection, separate estimates for persons with and without HIV infection indicate differences in the patient diagnostic rate and death rates between TB patients with and without HIV co-infection (6).

## Patient Diagnostic Rate, Case Detection Rate, and Program Effect

The quantitative relationship between the case detection rate, patient diagnostic rate, and expected program effect depends on the way we conceive case detection. Two approaches have been used in the past, perhaps best explained with the models of Styblo (model 1) (2,3) and Dye et al. (model 2) (4).

Model 1 assumes that cases are either detected after an average of 4 months or not at all (2,3). Patients whose cases are not detected either die or self-cure after an average of 2 years. Self-cure refers to patients reverting to latent infection without being treated. In model 2 (4), cases are detected at a certain rate (patient diagnostic rate), and the patients die or self-cure at a certain rate. The proportion of cases detected in model 2 thus depends on the relative size of these two rates: the larger the patient diagnostic rate, the larger the case detection rate and the shorter the average delay. As a result of these different assumptions, the same case detection rate of 70% is associated with a larger patient diagnostic rate and a larger impact on TB prevalence in model 2 than in model 1 (Appendix). In the absence of HIV infection, a case detection rate of at least 70% corresponds with a patient diagnostic rate of at least 0.84 per person-year in model 1 and a patient diagnostic rate of at least 1.17 per person-year in model 2.

How do these model targets compare with values of patient diagnostic rates we observe in the real world? A rough, indirect estimate of patient diagnostic rate in the Netherlands is 2.5 per person-year (Appendix). Of more relevance may be the direct estimates in countries with high rates of TB (Table): the patient diagnostic rate was 0.24 in China, 0.43 in Korea, and 0.51 in the Philippines. These three countries did not meet the goal for case detection by models 1 or 2.

**Table:** The patient diagnostic rate in China, Philippines, and Korea^a,b^

	Notification rate smear-+ TB per 100,000	Prevalence rate smear-+ TB per 100,000	PDR	Ref
China, 2000	17	72	0.24	10,11
Philippines, 1997	118	229	0.51	12,13
Korea, 1995	26	60	0.43	14,15

^a^TB, tuberculosis; +, positive; PDR, patient diagnostic rate; ref, reference number. ^b^In the Philippines, total prevalence was 310/100,000. Of 50 cases with drug susceptibility results and known treatment history, 37 (74%) had not been previously treated. The assumption was that 74% of prevalent smear-positive patients had not been previously treated. In Korea, total prevalence was 93/100,000. The prevalence of new smear-positive TB was obtained from the unpublished survey report.

For the patient diagnostic rate to be a useful indicator, the best reporting rate should be obtained. For instance, if general hospitals in China, or the private sector in the Philippines and Korea, fail to notify the patients they treat, the patient diagnostic rate will be underestimated (the same limitation applies to the case detection rate). Therefore, the use of patient diagnostic rate is not an alternative to a good reporting system but supports the development of such a system. If the notification system detects most cases (e.g., with a patient diagnostic rate exceeding the goal of model 2 of 1.17), then reporting data may be used exclusively to monitor trends, as is done in countries with low rates of disease.

## Limitation of the Patient Diagnostic Rate

A limitation of the patient diagnostic rate is that measuring TB prevalence is complicated and costly with the current standard methods, which require the use of mobile chest radiograph equipment as a screening tool. However, this limitation can be overcome. High standard prevalence surveys have been shown to be feasible (Table). Moreover, their cost represents a small proportion of the cost of control programs. TB control programs in the 22 countries with high rates of the disease annually cost an estimated U.S. $940 million, approximately half of which is within the TB program budget, while the other half represents health infrastructure costs (16). Twenty-two national surveys, performed with current standard methods once every 5–10 years, would cost approximately U.S. $25–$50 million in total, i.e., <U.S. $10 million per year. This cost represents at most 1% of the cost of TB control programs.

Nevertheless, new survey methods, using other diagnostic algorithms or new diagnostic methods, that do not require mobile chest radiographs would be beneficial. They would promote the measurement of TB case detection and program effect in the 22 countries with high rates of disease and in other high incidence-countries with limited resources, especially Africa.

## Conclusion

The patient diagnostic rate is a measurable indicator for detecting patients with previously untreated cases of smear-positive TB. The expected effect of a TB control program on transmission increases with an increasing value of this indicator. A patient diagnostic rate of >0.84 would correspond to the original WHO goal proposed by Styblo of detecting >70% of incident cases. A patient diagnostic rate of >1.17 would meet the goal of 70% case detection as used by Dye et al. to project the effect of the DOTS strategy (4). On the basis of further evidence about patient diagnostic rates and associated TB program impact, a revised goal may be formulated in the future.

While monitoring performance is extremely useful in the short-term, monitoring effects, or at least the trend of TB prevalence, is most important in the medium- and long-term. Programs aimed at reducing TB prevelance can assess whether the decrease is occurring through reporting rates, if case detection is good, or by carrying out prevalence surveys every 5–10 years, if the completeness of case detection varies or is uncertain. Prevalence surveys would provide direct information on indicator 23 for measuring progress towards meeting the Millennium Development Goals (1). Monitoring effect through prevalence surveys allows the patient diagnostic rate to be measured and the risk factors for nondetection to be identified by the health service. Developing new diagnostic methods, obviating the need for chest radiographs, would be extremely helpful for such surveys. Monitoring TB is recommended through prevalence surveys in countries with high rates of disease until reporting rates have been shown to provide sufficient information on TB trends in that particular setting.

## Appendix

### Model 1

**Figure A1:**
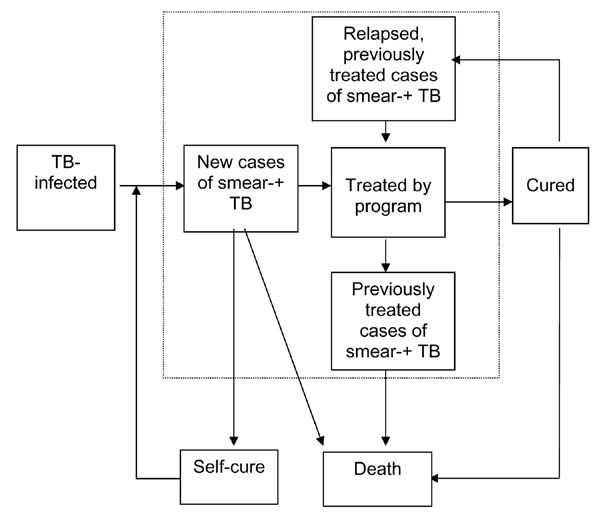
Model 1, used by Styblo, of tuberculosis case detection and treatment outcome in tuberculosis control program. Prevalent cases are those within dotted line.

Model 1, developed by Styblo (17,18), is presented in Figure A1. The case detection rate in model 1 is not a rate but a ratio: it does not reflect the speed at which cases are detected, but the proportion of incident cases detected. Model 1 assumed that, in the absence of treatment, the duration of the infectious period is 2 years. Each new self-reporting case was assumed to be detected after an average of 4 months. The case detection rate (the proportion of new cases detected) would thus directly determine the prevalence of new smear-positive tuberculosis (TB).

**Figure A2:**
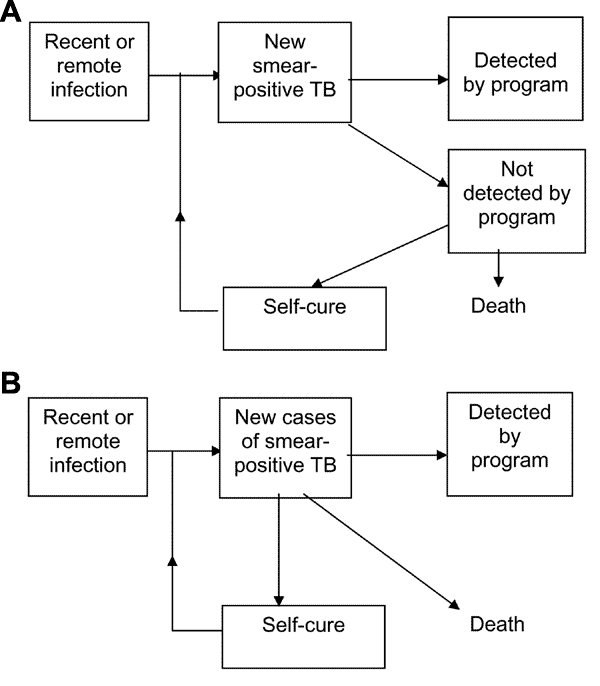
Models 1 and 2 on tuberculosis case detection. B) Model 2, used by Dye et al. with rate of case detection (PDR). B) the arrows depict rates; A) the arrows depict proportions of cases moving from one compartment to another.

Since the interest of this article is to assess case detection, the left part of Figure A1 is concentrated on, which is relevant for the prevalence of new cases of smear-positive TB only (Figure A2 A). When Figure A2 A and the assumptions above are used, the following expressions can be derived:



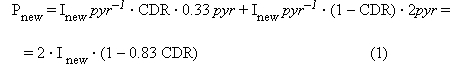



Where

P_new_ = prevalence ratio of new (i.e., never treated) cases of smear-positive TB

I_new_ = incidence rate (*pyr^–1^*) of new smear-positive TB

CDR= case detection rate = proportion of cases detected

By definition:



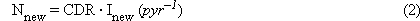



Where

N_new_ = notification rate (*pyr^–1^*) of new cases of smear-positive TB

and thus



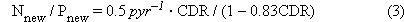



### Model 2

Model 2 was used by Dye et al. and assumes that incident cases are at risk for case detection and for death or self-cure (Figure A2 B) (modified from [19]). A similar approach is used by others (20). If the rates in model 2 were constant (i.e., independent of time since onset of disease), the combined rate of death and self-cure would be 0.5 *pyr^–1^* if the average duration of disease were 2 years in the absence of case detection. Indeed, Dye et al. assumed a rate of death of 0.3 *pyr^–1^* and a rate of self-cure of 0.2 *pyr^–1^* (19). The patient diagnostic rate (PDR) is defined as the rate at which patients are diagnosed. The proportion of incident cases detected (the case detection rate [CDR]) therefore equals:



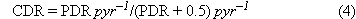



Which is equivalent to:



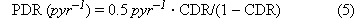



Since PDR may be estimated as N_new_/P_new_ this can also be presented as:



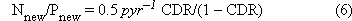



And since N_new_ = CDR ∙ I_new_:



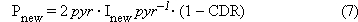



To assess to what extent a constant rate of detection (assumed by model 2) is supported by data on delay before diagnosis, we used data from the Netherlands Tuberculosis Register. From 1996 to 2002, a total of 468 new cases of smear-positive TB were diagnosed among the Dutch; these cases were found through passive case finding and had a recorded delay in treatment. Person-weeks at risk for detection were estimated by week since onset and used as the denominator for the rate of detection. Patient diagnostic rate was first estimated ignoring death rates and self-cure, and then by assuming an average rate of death and self cure of 0.5 *pyr^–1^*.

## Results

**Figure A3:**
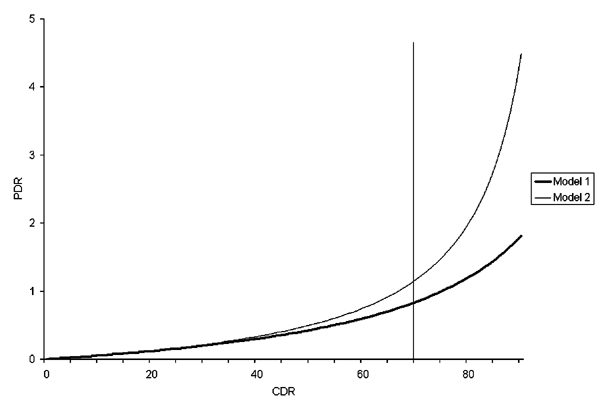
Relationship of case detection rate (CDR) and patient diagnostic rate (PDR) according to model 1 and model 2.

The relationship between case detection rate and patient diagnostic rate according to models 1 and 2 is presented in Figure A3. In both models, a one-to-one, nonlinear relationship exists between case detection rate and patient diagnostic rate: patient diagnostic rate increases with increasing case detection rates. This increase is steepest in model 2.

**Figure A4:**
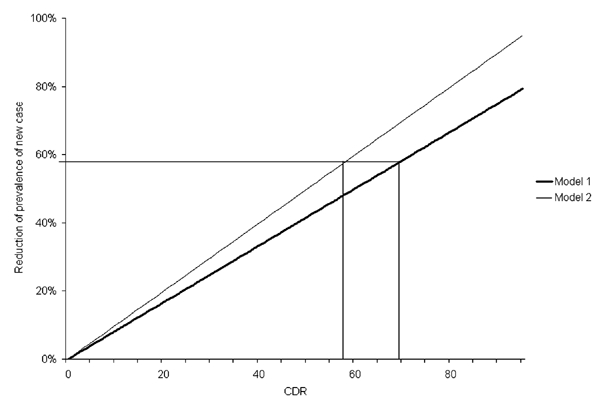
Reduction of prevalence of new smear-positive tuberculosis depending on the case detection rate (CDR) according to model 1 and model 2.

However, the same case detection rate in models 1 and 2 represent different effects on TB prevalence. For instance, a case detection rate of 70% according to model 1 (which is the basis of the current WHO goal) corresponds with a reduction of the prevalence of new cases of smear-positive TB of 58%. According to model 2, to achieve a 58% reduction of this prevalence, a case detection rate of 58% is required (Figure A4). If the goal is to reduce the prevalence of new cases of smear-positive TB by 58%, patient diagnostic rate would need to be 0.84, according to model 1, and 0.69 according to model 2. However, if the case detection rate goal is maintained at 70% while using model 2 (as was done by Dye et al. [3]), the corresponding patient diagnostic rate would be 1.17. Achieving this goal would be associated with a higher effect on TB prevalence than achieving the goal of 0.84 suggested by model 1.

**Figure A5:**
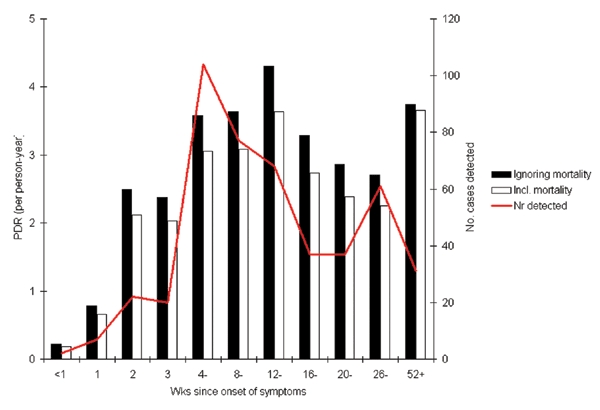
Estimates of the patient diagnostic rate (PDR) in the Netherlands, depending on the duration of symptoms.

In model 2, the patient diagnostic rate and the combined rate of death and self-cure were assumed to be constant, i.e., independent of time since diagnosis. The rate of detection based on reported patient's and doctor's delay in the Netherlands is presented in Figure A5. The rates of detection, first by ignoring and then by taking into account death and self-cure, were approximately 3.0 and 2.5 per person-year, respectively. The last figure corresponds with a case detection rate of 84%, according to expression (4). Patient diagnostic rate was lower during the first 4 weeks of disease. During the first 4 weeks, the rate increased approximately linearly from 0 to 2.5 per person-year.
