# Correction: Vol. 12, No. 10

**DOI:** 10.3201/eid1211.C11211

**Published:** 2006-11

**Keywords:** errata, correction, MaWhinney, 06-0019

In Human Prion Disease and Relative Risk Associated with Chronic Wasting Disease by Samantha MaWhinney et al., an error occurred in the list of references. Missing from the list is reference no. 36: Belay ED, Maddox RA, Gambetti P, Schonberger LB. Monitoring the occurrence of emerging forms of Creutzfeldt-Jakob disease in the United States. Neurology. 2003;60:176–81.

The corrected list of references appears in the online article at http://wwwnc.cdc.gov/eid/article/12/10/06-0019_article.htm

We regret any confusion this error may have caused.

